# Identification of Cassava MicroRNAs under Abiotic Stress

**DOI:** 10.1155/2013/857986

**Published:** 2013-11-14

**Authors:** Carolina Ballén-Taborda, Germán Plata, Sarah Ayling, Fausto Rodríguez-Zapata, Luis Augusto Becerra Lopez-Lavalle, Jorge Duitama, Joe Tohme

**Affiliations:** ^1^Agrobiodiversity and Biotechnology Project, International Center for Tropical Agriculture (CIAT), A.A. 6713, Cali, Colombia; ^2^Department of Systems Biology, Columbia University, 1130 Saint Nicholas Avenue, New York, NY 10032, USA; ^3^The Genome Analysis Centre, Norwich Research Park, Norwich NR4 7UH, UK

## Abstract

The study of microRNAs (miRNAs) in plants has gained significant attention in recent years due to their regulatory role during development and in response to biotic and abiotic stresses. Although cassava (*Manihot esculenta* Crantz) is tolerant to drought and other adverse conditions, most cassava miRNAs have been predicted using bioinformatics alone or through sequencing of plants challenged by biotic stress. Here, we use high-throughput sequencing and different bioinformatics methods to identify potential cassava miRNAs expressed in different tissues subject to heat and drought conditions. We identified 60 miRNAs conserved in other plant species and 821 potential cassava-specific miRNAs. We also predicted 134 and 1002 potential target genes for these two sets of sequences. Using real time PCR, we verified the condition-specific expression of 5 cassava small RNAs relative to a non-stress control. We also found, using publicly available expression data, a significantly lower expression of the predicted target genes of conserved and nonconserved miRNAs under drought stress compared to other cassava genes. Gene Ontology enrichment analysis along with condition specific expression of predicted miRNA targets, allowed us to identify several interesting miRNAs which may play a role in stress-induced posttranscriptional regulation in cassava and other plants.

## 1. Introduction

MicroRNAs (miRNAs) are short noncoding small RNA molecules that are transcribed in plants and animals and play key roles in posttranscriptional gene regulation [1]. Mature miRNAs are embedded into larger primary transcripts called pri-miRNAs and are released through a two-step cleavage process [2]. The first cleavage is performed by a Dicer homolog, called Dicer-like 1 (DCL1), which generates a stem-loop structure called precursor miRNA (premiRNA). Dicer makes a second set of cuts to produce the ~20–24 nt mature miRNA duplexed with a complementary miRNA*. The double-stranded fragment is exported to the cytoplasm where it dissociates and the mature ~21 nt miRNA is incorporated into the RNA-induced silencing complex (RISC). Guided by the miRNA sequence, the RISC complex down-regulates specific target genes either by cleaving or translational repression of their messenger RNAs [3–5].

In plants, regulation of gene expression by miRNAs is essential for normal growth and development, including leaf morphogenesis, patterning and polarity establishment, developmental timing, floral organ identity, and phytohormone signaling [3, 6]. Furthermore, miRNAs are also involved in plants' adaptation to biotic and abiotic stresses [7–10]. In the past decade, a large number of miRNAs have been discovered across several plant species; for instance, the miRBase database [11] contained 7,385 mature miRNA sequences for 72 plant species as of July 2013. The majority of these miRNAs have been validated using different computational and experimental approaches including deep sequencing, cloning, northern blots, and real time PCR [3, 12, 13].

Cassava (*M. esculenta* Crantz) is a crop widely grown as a staple food, animal feed, and as an industrial raw product in the tropical and subtropical regions of Latin America, Africa and Asia. Cassava is an important source of calories for more than half a billion people around the world and displays a unique ability to grow on low-fertility soils and tolerate drought conditions [14–16]. Despite the potential contribution of miRNAs to cassava improvement [17], molecular genetic information regarding cassava miRNAs remains sparse. Only recently, 153 cassava miRNAs were made available in miRBase (V.20) [11]. These miRNAs were obtained by Patanun et al. [18] using mostly computational techniques. In addition Pérez-Quintero et al. [19] recently analyzed small RNA libraries from cassava tissues infected and noninfected with *Xanthomonas axonopodis*, and Zeng et al. [20] made an study of conserved miRNAs in Euphorbiaceae family. A study of cassava miRNAs expressed under abiotic stress conditions is currently lacking. 

In this study, we characterize a population of cassava miRNAs obtained using next-generation sequencing of plants grown *in vitro* and in the field under heat and drought-like conditions. We compare current bioinformatics methods for miRNA discovery using high-throughput sequencing data and analyze strategies to increase the sensitivity of miRNA detection. We predict 881 cassava miRNAs and 1136 possible gene targets. We also validate the expression of 5 conserved miRNAs involved in heat and drought stresses predicted by our pipeline and suggest several interesting targets for future research.

## 2. Materials and Methods

### 2.1. RNA Sequencing and Processing of Raw Sequences

Total RNA was extracted from TAI16 cassava samples using TRIzol reagent (Invitrogen, USA) and treated with RQ1 RNase-free DNase (Promega, USA) according to the manufacturers' instructions. RNA quality was verified on agarose gels (28S : 18S > 1.5) followed by quantification in a NanoDrop spectrophotometer (Thermo Scientific, USA). DNA contamination was tested through PCR amplification of the 18S rRNA gene. RNA samples from different tissues/conditions were combined in equal concentrations into a single RNA pool [21] and a size-selected library (93–100 nt) representing adapter-ligated small RNAs was constructed and sequenced by synthesis using the Illumina Genome Analyzer (Illumina, University of Iowa DNA Facility).

 Raw sequences were processed as described by Sunkar et al. [22]. Quality trimming and adaptor removal were carried out using Cutadapt with error rate (–e) set to 0.1 [23]. The remaining reads were screened against ribosomal and transfer RNAs, snRNAs, and snoRNAs from Rfam [24] and TAIR [25] databases, and exact matches were removed from further analyses [21, 26]. Sequences, shorter than 20 nt and longer than 25 nt were also discarded [4]. Finally, exact copies of reads were collapsed while keeping track of the number of reads per unique sequence.

### 2.2. Bioinformatics Analysis of Collapsed Reads

To identify cassava miRNAs conserved in other plant species, a blast [27] search was performed (word size = 10) against plant mature miRNAs in miRBase (Release: 19.0, February 2013) [11]. We considered that a read was conserved when the alignment against miRBase had up to 1 mismatch and the read length was equal or larger than the length of the known miRNA. 

 Reads that did not satisfy the above criteria were considered candidate cassava-specific miRNAs and were analyzed to nominate true cassava miRNAs using the following analytical criteria: First, we used Bowtie (v1.0.0) [28] to identify perfect matches of these sequences to the cassava reference genome [29] (options: −*v*  0 to keep only matches with 100% sequence identity and −*a* to show all valid alignments; other parameters were kept at their default values). We only kept sequences with less than 30 hits as it has been previously reported that 29 sequences is the largest size of a miRNA gene family in crop plants including cassava, potato, and tobacco [18, 30, 31]. Second, we used miRDeep-P (v1.3) [32] (see Section 3) with a window size of 250 nt to identify precursor miRNA sequences (premiRNAs) in the cassava genome with a minimum score cut-off equal to −1 (log-odds probability of a sequence being a genuine miRNA precursor versus a background hairpin [33]). The identified premiRNAs that were used to predict secondary structures and folding energies using RNAfold [34] and the UEA sRNA toolkit [35]. To further improve the specificity of the dataset, we applied the following filters suggested by Meyers et al. [36] for plant miRNAs: (1) The miRNA and complementary miRNA sequences (miRNA*) had to be derived from opposite stem-arms forming a duplex with 2 nt 3′ overhangs; (2) base-pairing between the miRNA and miRNA* and had no more than 5 mismatches; and (3) There was at most one asymmetric bulge in the miRNA and miRNA* duplex and its length was 2 bp or less.

To evaluate the sensitivity and false positive rate (FPR) of miRDeep-P, MIReNA [37] and Mircheck [38] (see Section 3) random sets of 10,000 21-mers were retrieved from the *Arabidopsis thaliana* and *M. esculenta* reference genomes. To obtain each 21-mer, a random genome location was defined with uniform probability across all genome sites and the 21 nucleotides upstream from that position were retrieved. If any of the 21 positions in the genome sequences selected was not one of the 4 DNA nucleotides, the 21-mer was discarded (Supplementary script 1 available online at http://dx.doi.org/10.1155/2013/857986).

### 2.3. Characterization of miRNA Targets

PsRNA target [39] was used to screen all candidate miRNAs against 17,166 annotated genes in the cassava genome [29]. To minimize false positive predictions, the maximum expectation for complementarity was set at 2.0 and the length of the scoring region for complementarity at 17 [31]. Other parameters were set at their default values [38, 39]. Gene ontology (GO) annotations for predicted target genes were downloaded from phytozome [40]. GO enrichment analysis was performed using the singular enrichment analysis implemented in the AgriGO toolkit [41]. Statistical significance was calculated based on the hypergeometric test adjusted from multiple hypothesis testing. In the test, the hypergeometric distribution is used to compute the probability of obtaining the observed overlap in GO terms between two gene sets by chance [42]. For our analysis the first gene set—of size *n*—corresponds to candidate miRNA targets, and the second gene set—of size *N*—represents all cassava genes. If the first and second sets contain *k and K* genes annotated with a given GO term, respectively, then the probability of overrepresentation of said GO term is given by the following formula:

(1) $$\begin{array}{c} P_{\text{over}}\left(k\right)=\sum_{k^{\prime }=k}^{\min(K,n)}\frac{\left(\begin{array}{c} K\\ k^{\prime } \end{array}\right)\left(\begin{array}{c} N-\text{K}\\ n-k^{\prime } \end{array}\right)}{\left(\begin{array}{c} N\\ n \end{array}\right)},\\ \;\text{where}\;\left(\begin{array}{c} n\\ k \end{array}\right)=\frac{n!}{k!\left(n-k\right)!}. \end{array}$$

Correction for multiple hypotheses was done using the Benjamini-Hochberg-Yekutieli false discovery rate procedure [43].

### 2.4. Validation of Cassava miRNAs by Quantitative Real Time PCR (qRT-PCR)

Starting with 10 ng of total RNA from treated and nontreated plant material, total cDNA was synthetized in a 15 *μ*L reaction containing: 0.15 *μ*L 100 mM dNTPs (with dTTP), 1 *μ*L MultiScribe Reverse Transcriptase (50 U/*μ*L), 1.5 *μ*L 10x Reverse Transcription Buffer, 0.19 *μ*L RNase Inhibitor (20 U/*μ*L), 4.16 *μ*L Nuclease-free water, and 3 *μ*L stem-loop-specific miRNA primer 20X (TaqMan MicroRNA Reverse Transcription Kit, Applied Biosystems, USA). The tubes were incubated at 16°C for 30 min, 42°C for 30 min, 85°C for 5 min, and held at 4°C [44]. In order to use the 18S rRNA as the endogenous control for comparative Ct analyses [45], DNase-treated RNA samples were reverse transcribed to cDNA using SuperScript III Reverse Transcriptase and Random Primers (Invitrogen, USA) following the manufacturer's instructions. Following reverse transcription reactions, all cDNAs were diluted to 1 : 15 in nuclease-free water and stored at −20°C [46].

qRT-PCRs for each sample were carried out essentially as described by Fiedler et al. [46] in 10 *μ*L reaction volumes containing: 5 *μ*L TaqMan 2X Universal PCR master mix, 0.35 *μ*L 20X TaqMan Assays including miRNA-specific primers and TaqMan probes, 3.35 *μ*L H_2_O and 1.3 *μ*L of cDNAs (TaqMan MicroRNA Assays, Applied Biosystems, USA) [46]. The thermal cycling protocol consisted of incubation at 95°C for 10 min, followed by 40 cycles of 95°C for 15 s and 60°C for 60s. The sequences of the 18S rRNA-specific primers were as follows: Forward: 5′-atgataactcgacggatcgc-3′ (10 *μ*M) and Reverse: 5′-cttggatgtggtagccgttt-3′ (10 *μ*M). The target genes and 18S reference genes were amplified in parallel and in triplicate along with negative controls (not reverse transcribed and water blanks) [44–46]. 

Threshold cycle (Ct) values were automatically calculated by the instrument (Mastercycler ep realplex, software version 2.2. Eppendorf, USA). The relative expression of a sample *x* relative to a control *y* was analyzed using the 2^−ΔΔCt^ method with the following formula:

(2) $$\begin{array}{c} \text{Normalized}\;\text{fold}\;\text{change}=2^{-\Delta \Delta \text{Ct}},\text{with}\\ \Delta \Delta \text{Ct}={\left({\text{Ct}}_{\text{target}}-{\text{Ct}}_{18\text{SrRNA}}\right)}_{x}-{\left({\text{Ct}}_{\text{target}}-{\text{Ct}}_{18\text{SrRNA}}\right)}_{y}. \end{array}$$

## 3. Results

### 3.1. Deep Sequencing of Cassava miRNAs

**Figure 1:**
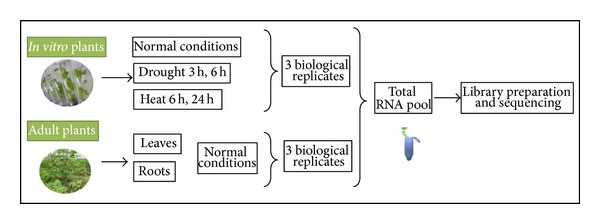
Source materials for RNA extraction, library preparation, and sequencing.

 A small RNA library was constructed from a two-month-old *in vitro* and adult TAI16 plants grown in the field for 9 months. Plants were subject to either no treatment, or heat and drought-like conditions as shown in Figure 1. In particular, drought stress was induced by taking *in vitro* plants out of the growth medium, washing their roots, and letting them rest at room temperature for 3 or 6 hours. Heat stress was induced by incubating *in vitro* plant materials at 37°C for 6 or 24 hours. Whole plants were used for RNA extraction in all *in vitro* samples, whereas total RNA was extracted from roots and leaves of field plants.

**Figure 2:**
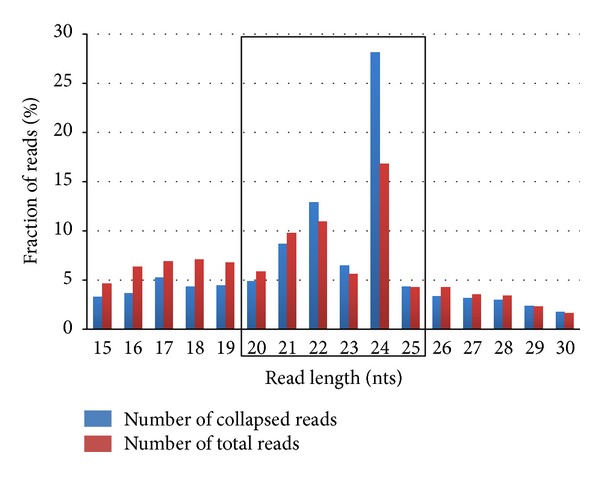
Frequency distribution of cassava small RNAs between 15 to 30 nt. Reads with 20 to 25 nts (black box) in length were selected for further analysis.

Deep sequencing of the pooled small RNA library yielded a total of 14,565,645 raw reads. Following the filtering procedures described in the methods section (Supplementary Table 1), we obtained 598,120 unique cassava small RNA sequences with sizes between 15 and 30 nt. The size distribution of this set of reads is shown in Figure 2. We observe the highest frequency peaks at 21, 22 and 24 nt, with 24 nt being the most frequent size of small RNAs in our library (~27%). The size distribution of cassava small RNAs is consistent with results observed in other plants using a deep-sequencing approach [21, 47–51]. Focusing on sequences with sizes between 20 and 25 nt, expected for most plant miRNAs, a preliminary set of 391,453 sequences was obtained.

Because many miRNAs have been found to be conserved across widely diverged plant species [52], we performed a sequence similarity search of our sequences against previously described plant miRNAs in the miRBase database [11]. Interestingly, 981 sequences in our library matched sequences from other species, suggesting that our experimental and analytical pipelines allowed us to identify conserved cassava miRNAs expressed under the tested conditions. We refer to these 981 sequences as conserved miRNA candidates to differentiate them from the remaining 390,472 candidate cassava-specific small RNA sequences. To narrow down the set of cassava-specific miRNAs and to validate the identity of conserved miRNAs, we aligned the conserved and nonconserved small RNA reads to the current assembly of the cassava genome [29]. This resulted in 146 (14.88%) conserved reads and 138,604 (35.50%) nonconserved reads that could be confidently mapped to the genome draft (Supplementary Table 1). These results confirm that these sequences are indeed present in the cassava reference genome and support their cassava-specific origin.

### 3.2. Comparison between miRDeep-P, Mirena, and Mircheck

Accurate identification of miRNAs from deep sequencing data is challenging. The general steps needed to characterize miRNAs typically involve the identification of premiRNA sequences followed by secondary structure predictions and the application of several filters to mitigate the occurrence of false positives. In order to find the most suitable analytical pipeline for our data, we compared three software packages Mircheck [38], MIReNA [37], and miRDeep-P [32]. This allowed us to decide, based on sensitivity and specificity, which tool had the best performance.

To evaluate sensitivity, we used two positive datasets: first, a set of 338 validated miRNAs from *A. thaliana *available through miRBase, second, the set of 981 conserved sequences found in our data. Although this second set has not been thoroughly validated, the fact that all sequences are conserved in other plant species lends confidence to the idea that most sequences in this dataset represent actual miRNAs. To evaluate specificity, we created two negative datasets using a custom script: first, a set of 10,000 random 21-mers from the *A. thaliana* reference genome, and second, a set of 10,000 random 21-mers derived from the *M. esculenta* reference genome (see Section 2). For each set of sequences, we calculated the percentage of predicted miRNAs divided by the number of sequences in the dataset. For the positive datasets this percentage is an estimation of the sensitivity of each method, for the negative controls it estimates the false positive rate (FPR). 

Table 1 shows the results of the analysis. In general, the sensitivity of all three methods was low (less than 10% in all cases). Specificity, on the other hand, was relatively high for miRDeep-P and Mircheck with a FPR close to 1% and very good for MIReNA, which did not predict any false positives. Since we could not detect any large differences in sensitivity between the three approaches and our aim was to obtain a comprehensive set of candidate cassava miRNAs, we decided to use the method that was most consistent between the two species. The fact that sensitivity shows a substantial drop for both Mircheck and MIReNA in the cassava datasets suggests that we could miss a significant number of miRNA candidates by using these programs. Moreover, adjusting various parameters of Mircheck and MIReNA related to the secondary structures, folding energies and base pairing of candidate premiRNAs we were unable to substantially increase sensitivity compared to results using the default parameters. For miRDeep-P however, we found that lowering the minimum score cut-off allowed a substantial improvement of sensitivity without a large drop in specificity (Supplementary Table  2). For this reason, we chose miRDeep-P for further analysis of miRNAs in cassava. 

The overall low sensitivity obtained in identifying the conserved cassava miRNA candidates is mostly related to the small (14.88%) fraction of sequences that align to the reference genome. Two possible factors that contribute to this result are (1) sequencing errors (both in the small RNA reads and in the reference genome) or single nucleotide variants between TAI16 and the reference genome line (AM560-2) and (2) the fact that the cassava genome is currently on an initial stage with 20 to 30% of sequences are predicted to be missing [29]. Despite these shortcomings, we could align 138,604 nonconserved reads, keeping the requirement for perfect matches.

### 3.3. Identification of Candidate Conserved and Nonconserved miRNAs

**Figure 3:**
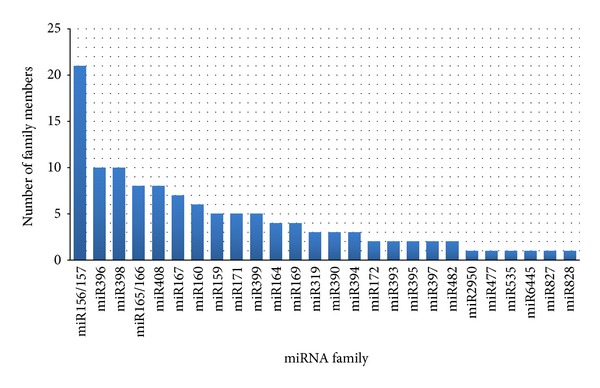
Distribution of 60 conserved cassava miRNAs grouped in 26 different families. Names correspond to homologous small RNAs in other plant species.

Based on the above method selection, calibration and filtering of the candidate miRNAs according to secondary structure criteria (see Section 2), we identified a final set of 118 conserved miRNA reads. These small RNA reads align to 106 potential precursors, and were grouped by miRDeep-P into 60 clusters on the basis of sequence similarity. We further joined these 60 clusters in 26 families (Figure 3) based on the names already established for corresponding miRNAs in other species. Performing the same analysis on the nonconserved small RNA reads, we obtained 821 candidate miRNA clusters mapping to 1,103 potential precursors. This completes our final set of 881 predicted cassava miRNA sequences which we make available as supplementary material (Supplementary file 1). 

### 3.4. Target Gene Identification and Functional Analyses

We identified 134 potential target genes for the 60 conserved miRNA sequences and 1,002 potential target genes for the 821 nonconserved sequences (Supplementary file 1). Interestingly, targets of conserved miRNAs not only included homologs of the corresponding targets in other species but also novel target genes. As expected, a substantial fraction (~41%) of conserved miRNAs targets are transcription-factors. For example, miR156 targeted genes in the *squamosa* promoter-binding family, miR159 targeted a MYB-like regulatory protein [17], and mir160 targeted several auxin response factors (ARF10, ARF16, and ARF17). Among newly predicted targets, miR166 was associated to a basic-leucine zipper (bZIP) transcription factor and miR169 to a basic helix-loop-helix (bHLH) DNA-binding protein. 

**Figure 4:**
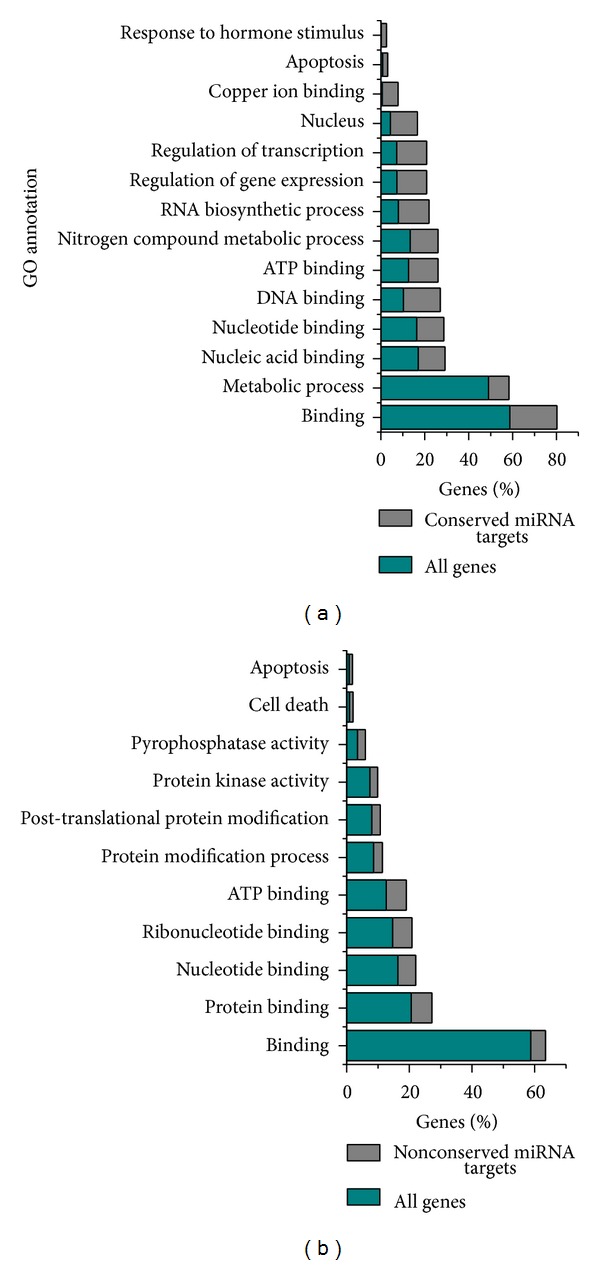
GO enrichment analysis of predicted miRNA targets. (a) Significantly overrepresented GO terms for conserved miRNAs identified in this study. (b) Significantly overrepresented GO terms for possible cassava-specific miRNAs. Cyan and gray bars indicate the fraction of miRNA targets and cassava genes annotated with a corresponding GO term, respectively. See Supplementary Tables 3 and 4 for the full list of significant terms.

Based on the GO annotation of the predicted targets, we searched for additional functional categories significantly enriched or depleted among target genes (conserved: Figure 4(a), nonconserved: Figure 4(b)). Statistically significant GO terms are reported in Supplementary Tables 3 and 4 for conserved and nonconserved target genes, respectively. Besides enrichment for transcription factors, represented by terms like DNA binding and transcription regulator activity, we found significant enrichment of processes related to cell death and signaling (Figure 4). These terms are associated to several predicted targets annotated as disease resistance proteins [53], trans-membrane receptors, or protein kinases/phosphatases. 

**Figure 5:**
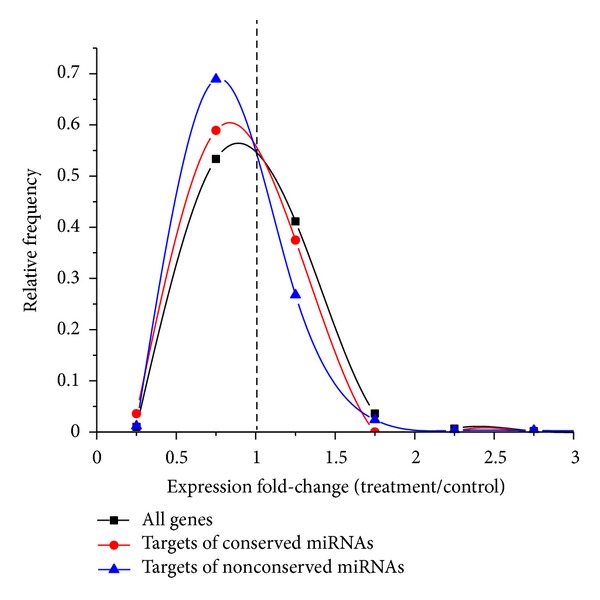
Relative expression levels of cassava genes after exposure to drought-like conditions. Data is from the study by Utsumi et al. [54]. Targets of conserved and nonconserved miRNA candidates display lower expression, on average, compared to all measured cassava genes upon stress treatment.

 As a further validation of predicted miRNAs and their targets, we analyzed the expression levels of TAI16 genes upon exposure of* in vitro* plants to drought-like conditions. Using the data recently published by Utsumi et al. [54], we found significantly lower fold-change expression values for the targets of conserved (Mann-Whitney *U* test *P* value: 0.05) and nonconserved (*P* value: 7.7 × 10^−16^) candidate miRNAs relative to about 20,000 genes represented in the microarray (Figure 5). Target genes of conserved miRNAs with the largest drop in expression included several proteins involved in redox balance such as an NAD(P)-linked oxidoreductase targeted by mir156, a laccase/diphenol oxidase targeted by miR397 and a potential respiratory burst oxidase targeted by miR399. However, not all target genes displayed lower expression upon stress treatment; chitinase A targeted by miR160, L-aspartate oxidase targeted by miR828, and an auxin-response family protein targeted by miR164 were among the genes with the largest increase in expression in the conserved set (see Supplementary file 1).

For the predicted targets of nonconserved miRNAs, we found a large (over 2-fold) expression drop for a few genes including homologs of BCL-2-associated athanogene 5, dihydroflavonol 4-reductase, and S-adenosyl-L-methionine-dependent methyltransferases. Overexpressed target genes included several proteins like gibberellin 2-oxidase, peroxidase, phenylalanine ammonia-lyase, ABA-induced PP2C phosphatase, and a couple of disease resistance protein homologs, which are associated with biotic and abiotic stresses (Supplementary file 1). Changes in the expression of these proteins support a possible role of miRNA sequences in response to oxidative stress, signaling, cell death, and metabolism, in agreement with the GO analysis.

### 3.5. Validation of Cassava Conserved miRNAs Related to Stress Response

To validate our bioinformatics analysis, we selected 5 conserved candidate miRNAs with *A. thaliana* homologs previously associated in stress response [20] (ath-miR156a [9, 55], ath-miR159a [9, 56], ath-miR160a [57], ath-miR397a [58, 59], and ath-miR408 [60, 61]). First, we verified that reads with identical sequences to these miRNAs were present in high copy numbers in our dataset. Indeed, for ath-miR156a, ath-miR159a, ath-miR160a, ath-miR397a and ath-miR408 we found 2,322, 22,117, 111, 1,081, and 2,216 reads, respectively. We also identified one to six precursors for each miRNA in the cassava reference genome. The minimal free energy for predicted secondary structures ranged from −37.60 to −92.8 kcal/mol (Supplementary Figure 1), which falls within the range found for other plants (−8.5 to −180.8 kcal/mol; average −65.05 kcal/mol [52]). Predicted precursor secondary structures (Supplementary Figure 1) were often identical or very similar to those previously reported by Amiteye et al. [17] and Patanun et al. [18]. 

**Figure 6:**
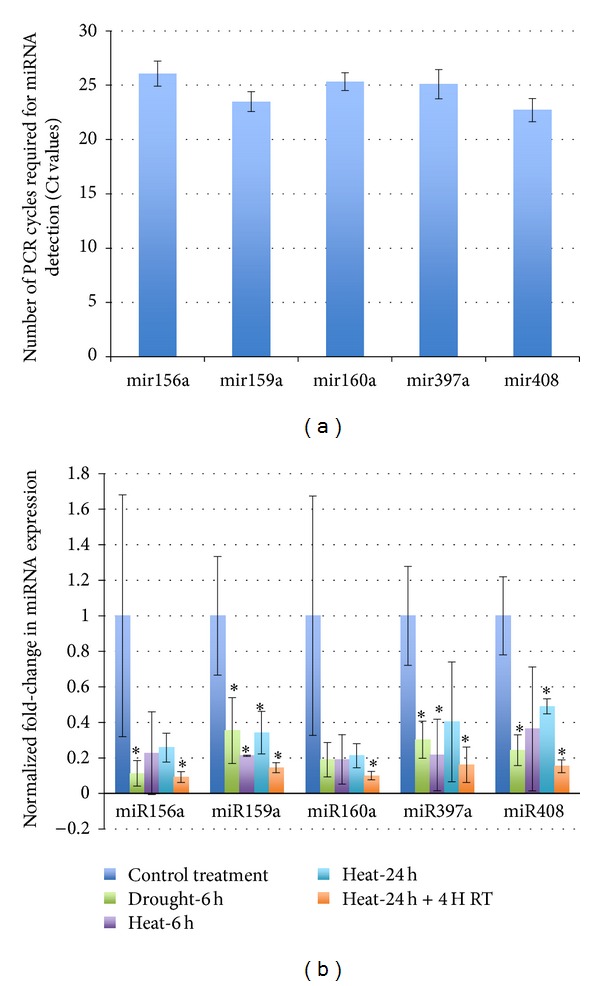
Validation of 5 predicted cassava miRNAs. (a) Mean cycle threshold (Ct) values showing the expression levels of the selected miRNAs under normal conditions; Ct values indicate the number of PCR cycles at which the amplification signal crosses a fixed threshold; lower Ct values correspond to higher expression levels. Error bars correspond to the standard deviation (SD) of Ct values (*n* = 3). (b) Comparison of the relative expression levels of the selected miRNAs under heat and drought treatments normalized to values in (a) using the 2^−ΔΔCt^ method. 18s rRNA was chosen as an endogenous control; error bars correspond to the normalized SD of 2^−ΔΔCt^ values (*n* = 3). *: Mann-Whitney *P* value <0.1; RT: Room temperature.

After establishing a high likelihood that these sequences were active in TAI16 plants, we quantified their expression under normal and stress conditions using a qRT-PCR approach. When no treatment was applied, we observed the amplification of all 5 sequences in less than 25 cycles (Figure 6(a)), indicating that the 5 miRNAs were expressed at roughly the same level; notably, Ct values above 35 are considered at the lower level of detection [62]. Looking at the expression values across different stress conditions (Figure 6(b)), we consistently found that miRNAs were expressed at lower level at either drought or heat stress conditions. Although *P* values for individual conditions were only marginally significant (<0.1), pooling the expression data for all conditions resulted in a significantly lower expression of miRNAs under stress compared to control conditions (*P* values between 0.01 and 0.04).

## 4. Discussion

The high adaptability of cassava to challenging environments [14] makes it a major food security crop in the light of deteriorating growth conditions associated with climate change. While multiple production challenges related to its nutritional value and disease resistance remain to be solved [63], understanding the physiology and genetic basis of drought and heat tolerance is of paramount importance for the improvement of this and other crops [64]. Interest in cassava miRNAs as potential targets for crop improvement has grown in recent years [17]; as demonstrated by a series of recently published research in this area [18–20]. Here, we make a contribution to these efforts by including, for the first time, stress-related conditions in the source materials for miRNA characterization. Compared to previous studies, we captured 7 additional conserved miRNA families relative to the study by Pérez-Quintero et al. [19], which was focused on biotic challenges, 8 additional conserved families compared to the study of Zeng et al. [20] that focused on conserved sequences across Euphorbiaceous plants, and 3 additional conserved families relative to the computational study by Patanun et al. [18]. Besides 26 conserved families, we tuned our bioinformatics pipeline in order to produce hundreds of additional cassava-specific miRNA candidates (Supplementary file 1). Additional filters, such as target gene identification, make it possible to use this resource to discover novel potential stress related small RNAs. 

We used a qRT-PCR approach to study the expression patterns of 5 conserved cassava miRNAs across different stress conditions. In agreement with previous studies [20], we found that stress-associated miRNAs can display significantly lower expression under heat and drought; this justifies our decision to also include untreated plant materials in the construction of the small RNA library. Interestingly, the average change in expression of predicted conserved and nonconserved miRNA targets in response to drought conditions indicates a significant down-regulation relative to other cassava genes (Figure 5). Thus, the regulatory mechanisms of miRNAs and their targets must be considered in a case by case basis. Targets of conserved miRNAs showed a significant enrichment of transcription factors (Figure 4(a)), an interesting example being miR160 which was previously associated to drought stress [65] and which we found to target several auxin response factors (AFR10, AFR16, and AFR17). AFR10 and AFR16 were shown to increase their expression in the roots of drought treated *Sorghum bicolor, *suggesting a common mechanism in cassava given our observation of lower miR160 expression under those conditions.

Multiple studies have found an association between miRNAs and plant stress responses (see, e.g., [66]). In order to relate our candidate cassava miRNAs with sequences from other species previously associated with stress we compared our set of conserved miRNAs to 1,085 miRNA sequences in the PASmir database [67]. PASmir is a database of miRNAs and associated target genes with roles in different plant abiotic stresses. Notably, out of 26 conserved miRNA families in our study, 22 have homologous sequences that were associated to stress in other species. While it is likely that the regulatory functions of these sequences are different in cassava compared to other plants, for 13 of these miRNA families we also found that their target genes were conserved in cassava according to our predictions (see Supplementary file 1). This suggests a common mechanism of miRNA-mediated regulation of stress responses for these families. An interesting example of a miRNA with potential roles in the stress response in cassava is miR164 which was predicted to regulate a NAC domain containing protein. NAC transcription factors have been reported to be induced by drought and to confer increased drought tolerance and sensitivity to ABA in transgenic *Arabidopsis* and rice plants [68, 69]. Another example is miR397 which we predict to target a laccase; this enzyme was previously associated with the response to water deprivation [70] and showed decreased expression levels when miR397 was up-regulated in sugarcane [71] and *Arabidopsis* [58].

Compared to the targets of conserved miRNAs, predicted targets of nonconserved sequences were mainly enriched in protein modification enzymes involving multiple kinases and phosphatases (Figure 4(b)). This suggests a possible link between miRNA regulation and cell signaling. Interestingly, one of our predicted novel miRNAs (sRNA001.00248394_x24_x2) targeted a protein phosphatase (cassava4.1_007913 m*|* PACid: 17981998) homologous to an abscisic acid (ABA) induced gene in *A. thaliana* (HAI3). ABA is an important plant stress hormone that is produced under drought conditions [72], making this sequence an interesting target for future studies. Another interesting target of included a gibberellin oxidase (cassava4.1_011871 m*|* PACid: 17968068) targeted by sRNA001.00535298_x24_x1, which was found to be overexpressed in MTAI16 plants subject to drought stress (Supplementary file 1) and was also differentially regulated under stress conditions in *Zoysia japonica* [73]. Also included among targets of nonconserved miRNAs was a phenylalanine ammonia-lyase (PAL) targeted by sRNA001.00341055_x20_x1. PAL has been shown to be differentially expressed following various abiotic stresses including drought in kenaf (*Hibiscus cannabis*) [74] and under water deprivation in maize [75, 76]; based on the Utsumi et al. [54] microarray data, PAL displayed a near two-fold increased expression following stress treatment in cassava plants (Supplementary file 1). As a final example, several WRKY transcription factors have been shown to be involved in plant drought and salinity stress responses [77]. Our analysis identified 6 different nonconserved miRNAs (Supplementary file 1) targeting members of the WRKY family; these included sRNA001.00283184_x21_x2 targeting WRKY33 which was previously shown to regulate genes with functions in the detoxification of reactive oxygen species in *Arabidopsis *[77]. 

## 5. Conclusions

We carried out a comprehensive screen of small RNA molecules across different growth conditions, tissues, and environmental stresses in cassava. The combination of next-generation sequencing and bioinformatics analyses allowed us to identify 60 conserved cassava miRNAs and hundreds of candidate small RNAs with potential roles in cassava's stress response. The joint analysis of identified sequences and their predicted targets provides a valuable tool for the prioritization of research objectives aimed at understanding cassava's posttranscriptional regulation network and its application in crop improvement. 

## Supplementary Material

Supplementary Materials: include two files. The first (PDF) file contains supplementary figure 1, suplementary tables 1 to 4 and suplementary script 1. The second (EXCEL) file contains the set of predicted conserved and non-conserved miRNAs identified in this study and their predicted target genes.

## Figures and Tables

**Table 1:** Comparison of miRDeep-P, MIReNA, and Mircheck using default parameters. miRNAs predicted by each of the three methods were filtered using criteria described by Meyers et al. [12]. FPR: False positive rate.

		*A. thaliana* positive set	*M. esculenta* positive set	*A. thaliana* negative set	*M. esculenta* negative set
miRDeep-P	Sensitivity	5.04%	4.89%		
	FPR			1.12%	0.99%
MIReNA	Sensitivity	6.52%	2.37%		
	FPR			0%	0%
Mircheck	Sensitivity	7.69%	0.82%		
	FPR			1.30%	0.03%
