# Supplementary material for: Identification of Cassava MicroRNAs under Abiotic Stress
Source: Int J Genomics. 2013 Nov 14;2013:857986. doi: 10.1155/2013/857986 (PMC3845235; doi:10.1155/2013/857986)
Supplement: Supplementary file 1 — Supplementary Materials: include two files. The first (PDF) file contains supplementary figure 1, suplementary tables 1 to 4 and suplementary script 1. The second (EXCEL) file contains the set of predicted conserved and non-conserved miRNAs identified in this study and their predicted target genes. [file 857986.f1.pdf]

Identification of Cassava MicroRNAs under Abiotic Stress

Carolina Ballén-Taborda[1], Germán Plata[2], Sarah Ayling[3], Fausto Rodríguez-Zapata[1], Luis Augusto Becerra Lopez-Lavalle[1], Jorge Duitama[1] & Joe Tohme[1]

[1]Agrobiodiversity and Biotechnology Project. International Center for Tropical Agriculture (CIAT). A.A. 6713, Cali, 76001000, Colombia. [2]Department of Systems Biology, Columbia University, 1130 St. Nicholas Ave. New York, NY 10032, USA. [3]The Genome Analysis Centre. Norwich Research Park. Norwich. NR4 7UH. UK.

SUPPLEMENTARY MATERIALS

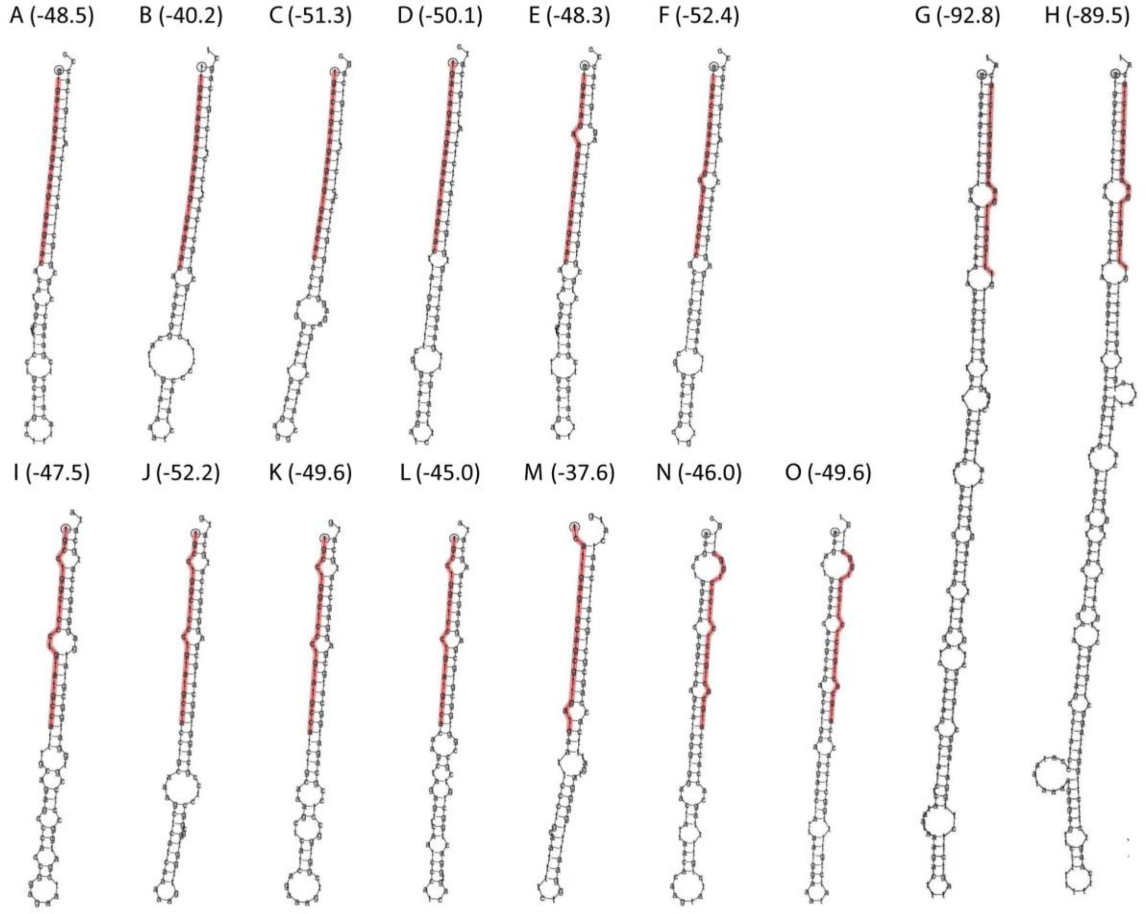

Supplementary figure 1. Secondary structure of the precursors of validated miRNAs: mes-miR156a (A, B, C, D, E, F), mes-miR159a (G, H), mes-miR160a (I, J, K, L), mes-miR397a (M) and mes-miR408 (N, O). The miRNA sequences are highlighted in red, folding free energies (kcal/mol) calculated with RNAfold [1] are shown in parentheses.

Supplementary table 1. Summary of the identification of cassava miRNAs.

Raw reads from deep-sequencing	14,565,645 Total Reads	
Adaptor and quality trimming	9,570,232 Total Reads	
Removal of rRNA, tRNA, snRNA, snoRNAs	598,120 Collapsed Reads<br>2,920,905 Total Reads	
20-25nt filtering	391,453 Collapsed Reads<br>1,553,668 Total Reads	
	**Conserved**	**Non-Conserved**
Total collapsed Reads	981	390,472
Aligned to the genome	146 (14.88%)	151,390 (38.77%)
Aligned to the genome at less than 30 loci	146	138,604
Number of precursors	338	441,567
Validated By MirDeep-P / clusters	114 / 64	12247 / 8325
Precursors Validated by Meyers 2008 Filter / clusters	106 / 60<br>(grouping 118 reads)	1103 / 821
Families of know miRNAs	26	--

Supplementary table 2. Sensitivity / Specificity analysis for different values of the minimum score parameter (-v) of miRDeep-P. Selected parameter is highlighted in blue.

**Score Cut-off**	***A. thaliana*** **Sensitivity (%)**	***A. thaliana*** **False Positive Rate (%)**	***M. esculenta*** **False Positive Rate (%)**
Default	5.04	1.12	0.99
10	0.00	0.00	0.00
5	4.45	0.00	0.00
0	6.53	1.12	0.99
**-1**	37.39	1.36	1.74
-5	39.47	1.37	1.82
-10	39.47	1.37	1.86
-25	39.47	1.37	1.86
-50	39.47	1.37	1.87
-80	39.47	1.37	1.88

Supplementary table 3. Significantly enriched GO terms among targets of candidate conserved cassava miRNAs. Results are based on the hypergeometric test corrected for multiple hypotheses (see Methods). The table is sorted by GO term type P: Biological process, F: Molecular function, C: Cellular component and by ascending FDR adjusted p-value.

GO term	Ontology	Description	Number of target genes	Number of total genes	p-value
GO:0032774	P	RNA biosynthetic process	42	1549	1.30E-06
GO:0045449	P	regulation of transcription	40	1402	1.30E-06
GO:0080090	P	regulation of primary metabolic process	40	1423	1.30E-06
GO:0060255	P	regulation of macromolecule metabolic process	40	1433	1.30E-06
GO:0031326	P	regulation of cellular biosynthetic process	40	1419	1.30E-06
GO:0010468	P	regulation of gene expression	40	1426	1.30E-06
GO:0019219	P	regulation of nucleobase, nucleoside, nucleotide and nucleic acid metabolic process	40	1408	1.30E-06
GO:0009889	P	regulation of biosynthetic process	40	1419	1.30E-06
GO:0051171	P	regulation of nitrogen compound metabolic process	40	1408	1.30E-06
GO:0051252	P	regulation of RNA metabolic process	40	1396	1.30E-06
GO:0006355	P	regulation of transcription, DNA-dependent	40	1396	1.30E-06
GO:0010556	P	regulation of macromolecule biosynthetic process	40	1419	1.30E-06
GO:0006350	P	transcription	42	1547	1.30E-06
GO:0006351	P	transcription, DNA-dependent	42	1545	1.30E-06
GO:0031323	P	regulation of cellular metabolic process	40	1451	1.50E-06
GO:0019222	P	regulation of metabolic process	40	1464	1.70E-06
GO:0016070	P	RNA metabolic process	43	1803	1.90E-05
GO:0006139	P	nucleobase, nucleoside, nucleotide and nucleic acid metabolic process	50	2280	3.10E-05
GO:0050794	P	regulation of cellular process	45	1970	3.10E-05
GO:0050789	P	regulation of biological process	45	1994	3.90E-05
GO:0065007	P	biological regulation	45	2093	0.00012
GO:0034645	P	cellular macromolecule biosynthetic process	47	2376	0.00047
GO:0009719	P	response to endogenous stimulus	5	39	0.00047
GO:0009059	P	macromolecule biosynthetic process	47	2379	0.00047
GO:0010033	P	response to organic substance	5	39	0.00047
GO:0009725	P	response to hormone stimulus	5	39	0.00047
GO:0006807	P	nitrogen compound metabolic process	50	2594	0.00049
GO:0010467	P	gene expression	46	2339	0.00055
GO:0044260	P	cellular macromolecule metabolic process	76	4740	0.0031
GO:0044249	P	cellular biosynthetic process	48	2906	0.013
GO:0009058	P	biosynthetic process	50	3122	0.019
GO:0043170	P	macromolecule metabolic process	77	5303	0.027
GO:0012501	P	programmed cell death	6	152	0.037
GO:0006915	P	apoptosis	6	152	0.037

GO:0005507	F	copper ion binding	15	126	8.60E-10
GO:0003677	F	DNA binding	52	2000	9.20E-07
GO:0016818	F	hydrolase activity, acting on acid anhydrides, in phosphorus-containing anhydrides	22	699	0.00032
GO:0016817	F	hydrolase activity, acting on acid anhydrides	22	712	0.00032
GO:0005524	F	ATP binding	50	2455	0.00044
GO:0032559	F	adenyl ribonucleotide binding	50	2520	0.00051
GO:0030554	F	adenyl nucleotide binding	52	2652	0.00051
GO:0001883	F	purine nucleoside binding	52	2652	0.00051
GO:0001882	F	nucleoside binding	52	2666	0.00051
GO:0017076	F	purine nucleotide binding	53	2996	0.0033
GO:0032555	F	purine ribonucleotide binding	51	2859	0.0033
GO:0032553	F	ribonucleotide binding	51	2859	0.0033
GO:0000166	F	nucleotide binding	55	3175	0.0037
GO:0003676	F	nucleic acid binding	56	3315	0.0054
GO:0005488	F	binding	154	11438	0.027
GO:0005634	C	nucleus	32	836	3.40E-08
GO:0043231	C	intracellular membrane-bounded organelle	32	1196	3.30E-05
GO:0043227	C	membrane-bounded organelle	32	1203	3.30E-05
GO:0043229	C	intracellular organelle	34	1803	0.0062
GO:0043226	C	organelle	34	1803	0.0062

Supplementary table 4. Significantly enriched GO terms among targets of candidate non-conserved cassava miRNAs. Results are based on the hypergeometric test corrected for multiple hypotheses (See Methods). The table is sorted by GO term type P: Biological process, F: Molecular function, C: Cellular component and by ascending FDR adjusted p-value.

GO term	Ontology	Description	Number of target genes	Number of total genes	p-value
GO:0008219	P	cell death	34	175	0.0036
GO:0016265	P	death	34	175	0.0036
GO:0006915	P	apoptosis	30	152	0.0045
GO:0012501	P	programmed cell death	30	152	0.0045
GO:0043412	P	macromolecule modification	194	1729	0.024
GO:0043687	P	post-translational protein modification	177	1556	0.024
GO:0006464	P	protein modification process	188	1674	0.025
GO:0032559	F	adenyl ribonucleotide binding	330	2520	2.40E-10
GO:0001883	F	purine nucleoside binding	336	2652	2.00E-09
GO:0001882	F	nucleoside binding	337	2666	2.00E-09
GO:0005524	F	ATP binding	314	2455	2.00E-09
GO:0030554	F	adenyl nucleotide binding	336	2652	2.00E-09
GO:0032555	F	purine ribonucleotide binding	344	2859	1.70E-07
GO:0032553	F	ribonucleotide binding	344	2859	1.70E-07
GO:0017076	F	purine nucleotide binding	350	2996	2.10E-06
GO:0043531	F	ADP binding	39	176	3.20E-06
GO:0005515	F	protein binding	449	4011	4.70E-06
GO:0000166	F	nucleotide binding	363	3175	7.20E-06
GO:0016887	F	ATPase activity	66	404	2.30E-05
GO:0017111	F	nucleoside-triphosphatase activity	96	661	2.30E-05
GO:0016462	F	pyrophosphatase activity	98	678	2.30E-05
GO:0016818	F	hydrolase activity, acting on acid anhydrides, in phosphorus-containing anhydrides	99	699	4.60E-05
GO:0016817	F	hydrolase activity, acting on acid anhydrides	100	712	5.60E-05
GO:0004672	F	protein kinase activity	163	1445	0.014
GO:0016773	F	phosphotransferase activity, alcohol group as acceptor	175	1608	0.041
GO:0042623	F	ATPase activity, coupled	37	251	0.041
GO:0042626	F	ATPase activity, coupled to transmembrane movement of substances	20	110	0.045
GO:0043492	F	ATPase activity, coupled to movement of substances	20	110	0.045

Supplementary script 1. Java script to retrieve random sequences from a genome. This script requires the NGSEP Java tools for analysis of Next Generation Sequencing data. http://sourceforge.net/projects/ngsep/

Usage:

java –Xmx500M RandomSeqs <Genome path> <Sequence length> <Sequence number>

```
--------------------------------------------

package org.cgiar.ciat.randomSeqs;

import java.io.FileOutputStream;
import java.io.IOException;
import java.io.PrintWriter;
import java.util.Random;
import net.sf.ngstools.genome.Genome;
import net.sf.ngstools.genome.GenomeAssembly;
import net.sf.ngstools.sequences.io.FastaSequencesHandler;

public class RandomSeqs {
	public static void main(String[] args) throws IOException {

		String fastaDir=args[0];
		GenomeAssembly genFile = new GenomeAssembly(fastaDir);
		int seqLenght=Integer.parseInt(args[1]);
		int numSeq=Integer.parseInt(args[2]);
		PrintWriter outFile = new PrintWriter(new FileOutputStream(args[3]));
		long START = 1;
		long END = genFile.getTotalLength();
		Random random = new Random();
		String seqRandom=null;
		int posRandom=0;

		for (int idx = 1; idx <= numSeq; ++idx){

			posRandom=showRandomInteger(START, END, random);
			seqRandom=genFile.getSequenceByAbsolutePosition(posRandom, seqLenght);
			if( seqRandom!=null&&!seqRandom.contains("N") ){
				outFile.println(">RandomSeq"+idx+"_"+"x21"+"_x1");
				outFile.println(seqRandom);
			}else {
				idx--;
			}
		}
		outFile.close();
	}
	private static int showRandomInteger(long aStart, long aEnd, Random aRandom){
		if ( aStart > aEnd ) {
			throw new IllegalArgumentException("Start cannot exceed End.");
		}
		long range = (long)aEnd - (long)aStart + 1;
		long fraction = (long)(range * aRandom.nextDouble());
		int randomNumber =  (int)(fraction + aStart);
		return randomNumber;
	}
}
```

References

1. Hofacker IL, Fontana W, Stadler PF, Bonhoeffer LS, Tacker M, Schuster P: **Fast folding and comparison of RNA secondary structures**. *Monatshefte für Chemie* 1994, **125**(2):167-188
